# Supplementary material for: HIVprotI: an integrated web based platform for prediction and design of HIV proteins inhibitors
Source: J Cheminform. 2018 Mar 9;10:12. doi: 10.1186/s13321-018-0266-y (PMC5845081; doi:10.1186/s13321-018-0266-y)
Supplement: Supplementary file 2 — Additional file 2. Source code of HIVProtI web server. [file 13321_2018_266_MOESM2_ESM.zip › HIVprotI_Source-code/hivprotI/weka-3-6-6/doc/weka/classifiers/bayes/NaiveBayes.html]

NaiveBayes


---


|  |  |  |
| --- | --- | --- |
| **Overview** \| **Package** \| **Class** \| **Tree** \| **Deprecated** \| **Index** \| **Help** \| **Weka's home** | |  |
| **PREV CLASS**   **NEXT CLASS** | **FRAMES**    **NO FRAMES**     **All Classes** |  |
| SUMMARY: NESTED \| FIELD \| CONSTR \| METHOD | DETAIL: FIELD \| CONSTR \| METHOD |  |


---


## weka.classifiers.bayes Class NaiveBayes

```
java.lang.Object
  weka.classifiers.Classifier
      weka.classifiers.bayes.NaiveBayes
```

**All Implemented Interfaces:**: java.io.Serializable, java.lang.Cloneable, CapabilitiesHandler, OptionHandler, RevisionHandler, TechnicalInformationHandler, WeightedInstancesHandler

**Direct Known Subclasses:**: NaiveBayesUpdateable

---

``` public class NaiveBayes extends Classifier implements OptionHandler, WeightedInstancesHandler, TechnicalInformationHandler ```

Class for a Naive Bayes classifier using estimator classes. Numeric estimator precision values are chosen based on analysis of the training data. For this reason, the classifier is not an UpdateableClassifier (which in typical usage are initialized with zero training instances) -- if you need the UpdateableClassifier functionality, use the NaiveBayesUpdateable classifier. The NaiveBayesUpdateable classifier will use a default precision of 0.1 for numeric attributes when buildClassifier is called with zero training instances.
